# Chemical Composition, Antimicrobial, Antioxidant, and Antiproliferative Properties of Grapefruit Essential Oil Prepared by Molecular Distillation

**DOI:** 10.3390/molecules25010217

**Published:** 2020-01-05

**Authors:** Weihui Deng, Ke Liu, Shan Cao, Jingyu Sun, Balian Zhong, Jiong Chun

**Affiliations:** National Navel Orange Engineering Research Center, College of Life Sciences, Gannan Normal University, Ganzhou 341000, China; dwh110by@163.com (W.D.); liuke121602026@126.com (K.L.); scoral29116@163.com (S.C.); SJYnj_1997@163.com (J.S.); bal.zh@163.com (B.Z.)

**Keywords:** grapefruit essential oil, molecular distillation, GC-MS, antimicrobial, antioxidant, anticancer activity

## Abstract

Grapefruit essential oil has been proven to have wide range of bioactivities. However, bioactivity of its molecular distillate has not been well studied. In this study, a light phase oil was obtained by molecular distillation from cold-pressed grapefruit essential oil and GC-MS was used to identify its chemical composition. The antimicrobial activity of the light phase oil was tested by filter paper diffusion method, and the anticancer activity was determined by the Cell Counting Kit-8 (CCK-8) assay. Twenty-four components were detected with a total relative content of 99.74%, including 97.48% of terpenes and 1.66% of oxygenated terpenes. The light phase oil had the best antimicrobial effect on *Bacillus subtilis*, followed by *Escherichia coli*, *Staphylococcus aureus* and *Salmonellaty phimurium*. DPPH and ABTS assays demonstrated that the light phase oil had good antioxidant activity. The CCK-8 assay of cell proliferation showed that the light phase oil had a good inhibitory effect on the proliferation of HepG2 liver cancer cells and HCT116 colon cancer cells.

## 1. Introduction

With the frequent occurrence of food safety issues and the toxicity of synthetic chemicals, the demand for safe and natural alternatives is growing. Plant extracts have been used since ancient times, and now the focus is on their role in health promotion and their treatment and prevention properties for various diseases. In the past few decades, plant essential oils (EOs) have attracted a lot of interest due to their safety and pharmacological properties including bacteriostatic, free radical scavenging, anti-inflammatory, and inhibitory effects on malignant tumor cell proliferation [1,2,3,4]. Citrus Eos are the main aromatic by-products of the juice extraction industry and are widely used in food, cosmetics and pharmaceutical industry [5,6,7]. The annual global production of citrus EO is approximately 16,000 tons, and the cost is about $14,000/ton on the international market. Thus, citrus EO is of great demand and is one of the more promising market prospects [8]. Grapefruit (*Citrus paradisi* Macf.), one of the world′s largest production citrus families [9], is famous for its taste and nutritional value. Grapefruit EO is extracted from grapefruit peel and has been used for a long time as a valuable ingredient for its characteristic aroma in flavor and fragrance [10,11]. Similar to most citrus EOs, its major components are terpenes and terpene oxides. Terpene oxides include alcohols, ethers, aldehydes, ketones, and esters [12,13,14], which are the main source of the aroma, whereas terpenes contribute less to the aroma. In spite of extensive studies on the aroma features of grapefruit EO, in recent years, more and more researchers have become interested in exploring their biological and pharmacological activities. Grapefruit EO has been reported to have a wide range of bioactivities. It was shown to inhibit the growth of food-borne spoilage bacteria and pathogenic strains [15,16,17]. Okunowo et al. (2013) found that grapefruit EO obtained from the peel by hydrodistillation exerted inhibitory effects against bacteria and fungi, andmay be further developed for the treatment of certain diseases [18]. Grapefruit EO has shown antioxidant activity, which was important for food preservation and disease prevention [19,20]. Ahmed et al. (2019) reported that grapefruit EO extracted by hydrodistillation had antioxidant activity by using DPPH and FRAP assays [21]. Grapefruit peel extracts have been shown to decrease the HL-60 cell viability in a concentration-dependent manner [22]. In fact, grapefruit extracts (a mixture of EO and other nonvolatile phytochemicals) could also inhibit the growth and proliferation of cancer cells such as neuroblastomas, leukemias, and prostate and lung cancer lines [23,24,25]. Cuthrell et al. (2006) reviewed the anticancer activities of phytochemicals found in grapefruit [26].

Most grapefruit EO samples used for bioactivity studies were made by cold-pressing, steam distillation, or hydrodistillation methods. Cold-pressing is the predominant method to extract most citrus peel EOs, including grapefruit EOs. In commercial practice, grapefruitis processed to obtain juice and other by-products. EO is one of the primary grapefruit by-products. Large-scale grapefruit EO is mainly prepared by a cold-pressing method based on John Bean FoodTech (JBT) juice extractors and its technology, which is used by 75% of the world’s citrus juice production [27]. The juice and EO are extracted separately and simultaneously. The EO was extracted by mechanical rupturing of the oil sacs in the flavedo, expressing the oil as an aqueous emulsion from which it is separated by centrifuging. The EO recovery is a physical separation process and no heat is applied throughout the whole extraction procedure. The operation temperature is much lower than in the distillation procedure. Thus, the EOs will have characteristics that are closer to those of the essence present in the grapefruit matrix. Large scale production, low cost and the aroma characteristic remaining are the big advantages of the cold-pressing method. However, cold-pressed grapefruit oil contains waxes, pesticide residues, coumarins, carotenoids and other nonvolatile components [28,29,30], some of them also have good bioactivities that may cause bias in bioactivity research of EO. César et al. (2009) found that furanocoumarins isolated from grapefruit peel oil showed potent in vitro inhibitory activity against intestinal cytochrome P450 3A4, an enzyme involved in “grapefruit/drug” interactions in humans [28]. Steam distillation or hydrodistillation was carried out at relatively high temperature which may cause degradation of some thermal sensitive molecules [18,31]. To avoid such problems, and find a new way to use the commercial available cold-pressed grapefruit EO in biochemistry and pharmachutical fields, we used a molecular distillation method to prepare grapefruit oil samples for our bioactivity tests.

Molecular distillation is a special liquid–liquid separation technology under high vacuum, which is employed as a separation process in the food industry [32,33]. Molecular distillation can divide the EO mixture into two different phases according to the free path of different molecules at low temperature. Molecular distillation is prominent with the advantages of low temperature treatment and high vacuum application, which is very suitable for thermolabile compounds and is used for concentrating and refining EOs [34,35]. At present, there are seldom reports on the antibacterial and anticancer activity of Eos obtained by molecular distillation. In this study, the cold-pressed grapefruit EO was processed by molecular distillation technology and the light phase essential oil (LPEO) was collected. Its constituents were identified by GC-MS. The activities of LEPO were tested on microorganisms and malignant proliferating cells (HCT116 colon cancer cells and HepG2 liver cancer cells) were tested. We expect that this work can stimulate the development of new agents for food preservation and chemo-preventive anti-cancer treatments.

## 2. Results and Discussion

### 2.1. Chemical Composition of the Light Phase Grapefruit Essential Oil

**Figure 1:**
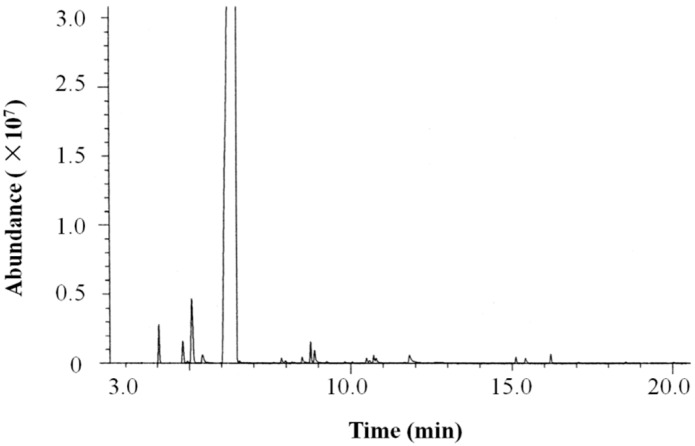
Total ion chromatogram of grapefruit light phase essential oil (LPEO).

The chemical composition of the grapefruit light phase essential oil (LPEO)was analyzed by GC-MS. The total ion chromatogram (TIC)of LPEO is shown in Figure 1. The relative content of each component was calculated by the peak area normalization method. The components were identified according to retention index and the NIST mass spectral library.

As shown in Table 1, twenty-four compounds, accounting for 99.74% of the total oil were identified. Monoterpenes were the major components, accounting for 96.93% of the total oil. Limonene (93.33%) was the predominant component of monoterpenes, followed by β-myrcene (2.16%), α-pinene (0.76%), and sabinene (0.60%). Monoterpene oxide (1.62%) included carvone (0.41%), cis-limonene oxide (0.43%), and trans-limonene oxide (0.33%). The sesquiterpene (0.55%) included caryophyllene (0.20%), β-cubebene (0.14%), α-copaene (0.13%), etc. Caryophyllene oxide (0.04%) was the only sesquiterpene oxide detected. In addition, three linear aldehydes: Octanal (0.36%), decanal (0.19%), and nonanal (0.05%) were found in LPEO. Pino et al. (1999) reported the chemical composition of grapefruit EO prepared by steam distillation from solids and effluents produced during commercial oil extraction [31]. The limonene content (70.9%) in steam-distilled oil was much less than LPEO (93.33%); however, the content of myrcene (13.6%) and α-pinene (3.8%) was much higher than LPEO (myrcene 2.16% andα-pinene 0.76%). Also, Okunowo et al. (2013) reported the components of grapefruit EO obtained by hydrodistillation [18]. The content of limonene (75.07%)was closed to that of steam-distilled oil. Cold-pressed grapefruit oil was shown to have a limonene content of 93.47%, however, the corrected limonene content became 85.60% when nonvolatiles were excluded [36]. The composition of distilled samples of grapefruit EO still vary from each other according to genetic differences, soil type, maturity stages, weather types and culturing conditions etc [37].

### 2.2. Antimicrobial Activity

Grapefruit EOs prepared by cold-pressing or hydrodistillation using a Clevenger-type apparatus have shown a wide spectrum of antimicrobial activity in vitro [18,37]. However, antimicrobial activity of grapefruit EO prepared by molecular distillation has not been well studied. We tested LPEO on five microorganisms and the results obtained are shown in Table 2. The filter paper diffusion method was used to test the antibacterial activity of LPEO against different bacteria, and the activity of LPEO was evaluated according to the diameter of the inhibition zone and the minimum inhibitory concentration (MIC) values. LPEO exhibited strong antibacterial effects on the four bacteria tested. From the scale of the inhibition zone, LPEO had the strongest inhibitory effect on *B. subtilis* with a maximum diameter of 35.59 mm, followed by *E. coli*, *S. aureus*, and *S. typhimurium*. LPEO had no inhibitory activity against *P. aeruginosa* with an inhibition zone of 8.57 mm. *P. aeruginosa* belongs with *E. coli* and *S. typhimurium* to the group of Gram-negative bacteria but exhibits quite a different response to LPEO. This phenomenon may be partly due to its relatively low outer membrane permeability. LPEO molecules enter the periplasm by diffusion through the channels of nonspecific porins in the outer membrane, and this pathway in *P. aeruginosa* is 10- to 100-fold less efficient than that in *E. coli* [38]. Regarding MIC values, LPEO showed the best antimicrobial activity against *Bacillus subtilis* with a MIC value of 0.78 µL/mL. Based on the inhibition zone and MIC values, the order of sensitivity of the different bacteria was: *B. subtilis* > *E. coli* > *S. aureus* > *S. typhimurium* > *P. aeruginosa*. Since cold-pressed grapefruit oil (*Citrus paradisi* Macf.) has been evaluated as “generally recognized as safe” (GRAS) by the Expert Panel of the Flavor and Extract Manufacturers Association (FEMA) [39], and LPEO, a distillate from cold-pressed EO, showed strong sensitivity to most tested microorganisms, it appeared to be suitable to food applications. These results demonstrate that molecular distillation technology can provide a grapefruit EO fraction with good antimicrobial activity.

Uysal et al. (2011) evaluated the antibacterial activities of grapefruit Eos from solvent-free microwave extraction (SFME) and hydrodistillation (HD) by the disc-diffusion method [17]. The Eos obtained from SFME and HD showed the highest activity against *S. aureus* with inhibition zones of 53 and 41 mm, respectively, higher than LPEO (24.34 mm). The activity against *E. coli* (30 mm and 28 mm) was close to our result (26.86 mm). Both of their samples and LPEO showed no obvious activity against *P. aeruginosa.* LPEO showed better activity against *S. typhimurium* (21.70 mm) than their samples (15 mm and 13 mm). Although a lot of plant EOs have shown antimicrobial activity, the reason of this capacity is not well known. It could be provoked by the major components of the EOs or due to a synergistic effect among the major components and the minor ones. Different preparation methods yield EO samples with differences in chemical composition and relative content, and cause differences in antimicrobial activity.

### 2.3. Antioxidant Activity

A lot of EOs have been reported to scavenge the free radicals that cause damage to the body and reduce the risk of many diseases originating from oxidative stress. In order to measure the effect of LPEO and determine its potential application in food, cosmetic or pharmaceutical industries, we evaluated its antioxidant activity using two different assays: The 2,2-diphenyl-1-picrylhydrazyl (DPPH)and 2,2′-azino-bis(3-ethylbenzthiazoline-6-sulfonic acid) radical (ABTS) assays. Butylated hydroxytoluene (BHT) was used as positive control. The *IC*_50_ values of BHT in DPPH and ABTS were 0.03 mg/mL and 0.01 mg/mL, which was consistent with the literature [40]. The DPPH and ABTS activities of LPEO were obtained with *IC*_50_ values of 22.06 ± 0.92 mg/mL and 15.72 ± 0.32 mg/mL, respectively. LPEO had better antioxidant activity than cold-pressed grapefruit EO in the DPPH assay (*EC*_50_ > 40 mg/mL) and hydrodistilled grapefruit EO in the ABTS assay (*EC*_50_ = 27.5 mg/mL) [35]. Compared with cold-pressed orange oil, LPEO had much lower antioxidant activity in the DPPH assay (*IC*_50_ = 3.01 ± 0.20 mg/mL) and better activity in the ABTS assay (*IC*_50_ = 23.25 ± 0.84 mg/mL) [41].

### 2.4. Antiproliferative Activity of LPEO in HepG2 and HCT116 Cancer Cells

**Figure 2:**
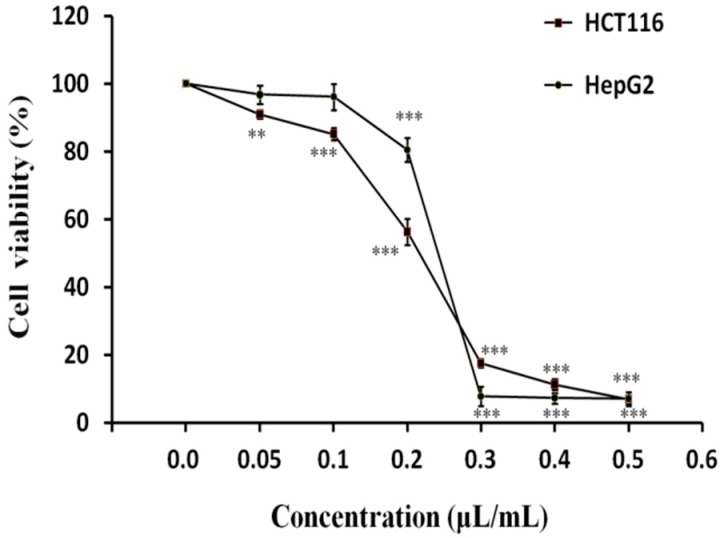
Effects on the viability of cancer cellsHepG2 and HCT116 as a function of LPEO concentration. Significant decreases in cell viability of cancer cells are seen at increasing LPEO concentrations compared to untreated controls (control group was set to 100%). **—Very significant at *p* < 0.01, ***—Highly significant at *p* < 0.001.

The effects of different concentrations of LPEO on the proliferation of HepG2 liver cancer and HCT116 colon cancer cells were tested by the Cell Counting Kit-8 (CCK-8) method [42,43]. The results are shown in Figure 2. The viability rate of both cell types decreased with increasing LPEO concentration. When the concentration of LPEO was less than 0.1 μL/mL, no obvious change of viability of HepG2 cells was observed. However, when the concentration of LPEO was higher than 0.1 μL/mL, the viability of HepG2 cells significantly decreased; at the LPEO concentration of 0.3 μL/mL, the viability was 7.4%only. LPEO also had a good inhibitory effect on the growth of HCT116 colon cancer cells. At the concentration of0.05 μL/mL or higher, the viability of HCT116 cells significantly decreased. It was as low as 7.43% when the concentration of LPEO was 0.5 μL/mL. GraphPad Prism™ (Version 5.00) software (GraphPad Software, San Diego, CA, USA) was used to calculate *IC*_50_ values. *IC*_50_ value of HepG2 and HCT116 was 0.24 and 0.20 μL/mL, respectively. These results indicate that LPEO has a significant inhibitory effect on the proliferation of HepG2 hepatoma cells and HCT116 colon cancer cells in vitro.

Sun et al. (2002) studied antiproliferative activity of grapefruit fruit extract on the growth of HepG2 human liver cancer cells in vitro [22]. The extract showed antiproliferative activity in a dose-dependent manner with the median effective dose (*EC*_50_) value of 130.09 mg/mL. However, they did not identify the specific phytochemicals which were responsible for antiproliferative activity. Manassero et al. (2013) studied the antiproliferative activity of cold-pressed EO from mandarin peel and its principal component limonene [44]. Mandarin EO and limonene exhibited *IC*_50_ of 0.063 μL/mL and 0.150 μL/mL against HepG2 cells, respectively. The much higher activity of mandarin EO than LPEO (0.24 μL/mL) may attributed to other high potent phytochemicals in cold-pressed EO. We have reported antiproliferative activity of the ‘Gannanzao’ orange EO (GOEO) prepared by hydrodistillation, which exhibited *IC*_50_ of 0.29 μL/mL and 0.35 μL/mL against HepG2 cells and HCT116 colon cancer cells, respectively [43]. LPEO showed a slightly higher activity than GOEO, which may be attributed to its higher limonene content (LPEO 93.33%, GOEO 88.07%).

The discussion about anticancer activity of some EO components has been made by Mukhtar et al. [45]. Our study preliminarily tested the inhibitory effect of LPEO on the proliferation of HepG2 liver cancer cells and HCT116 colon cancer cells. The anticancer activity of LPEO and its components on cancer cells and their mode of action deserve further study.

## 3. Materials and Methods

### 3.1. Materials

Cold-pressed Marsh white grapefruit (*Citrus paradisi* Macf., Lakeland, FL, USA) EO was purchased from Ungerer Limited. 2,2-diphenyl-1-picrylhydrazyl (DPPH) was purchased from Tokyo Chemical Industry Co., Ltd. (Tokyo, Japan), 2,2′-azino-bis(3-ethylbenzthiazoline-6-sulfonic acid) (ABTS), *n*-alkanes(C8–C20)were purchased from Sigma-Aldrich (St. Louis, MO, USA). Butylated hydroxytoluene (BHT) was purchased from Macklin, Shanghai, China. The following microorganisms were purchased from Beijing, China General Microbiological Culture Collection Center (CGMCC): *Escherichia coli* (ATCC25922), *Staphylococcus aureus* (ATCC25923), *Bacillus subtilis* (ATCC6633), *Salmonella typhimurium* (ATCC14028), and *Pseudomonas aeruginosa* (ATCC9207).

### 3.2. Preparation of Grapefruit Light Phase EO Sample

Grapefruit light phase EO(LPEO) was obtained by molecular distillation from cold-pressed grapefruit EO (*Citrus paradisi* Macf., Lakeland, FL, USA) using a wiped-film molecular distillation apparatus (Pope Two Inch Laboratory Scale Wiped-Film Molecular Still & Evaporator, Pope Scientific Inc., Saukville, WI, USA). The evaporation temperature and operation pressure were 55 °C and 6.0 Torr, respectively. Cold-pressed grapefruit EO was fed at room temperature and the feeding rate was 3.0 mL/min. The rotational speed of the roller wiper (Pope Scientific Inc., Saukville, WI, USA) was 325 rpm, and the condenser temperature was 0 °C. The final grapefruit EO sample (LPEO) was obtained from the light phase outlet with the yield of 86%.

### 3.3. GC-MS Analyses

The constituents of LPEO were analyzed using an Agilent 7890B gas chromatograph coupled with an Agilent mass spectrometer detector (Agilent Technologies, Santa Clara, CA, USA). The GC was equipped with a HP-5 column (30.00 m × 0.25 mm × 0.25 µm). Mass spectra were obtained by electron ionization (EI) at 70 eV. The injector and detector were operated at 250 °C and 300 °C, respectively. The temperature program was 80 °C for 4 min, and then increased at 5 °C/min to 250 °C and held constant for 10 min. The constituents were identified by comparing their mass spectra with the National Institute of Standards and Technology (NIST, version 2010, U.S. Department of Commerce, Gaithersburg, MD, USA) data reference. The retention indices (RI) of the constituents were determined by adding a C8–C20 *n*-alkanes mixture to the essential oil before injecting in the GC-MS equipment and analyzing it under the same conditions described above.

### 3.4. Antimicrobial Activity Assays

#### 3.4.1. Microbial Growth Conditions

The microbial strains were maintained in nutrient agar media at 37 °C. Subsequently, one colony from each culture was inoculated in liquid medium for 18–24 h with shaking (200 rpm) to obtain freshly cultured microbial suspensions (>10^8^ CFU mL^−1^) for test.

#### 3.4.2. Determination of Diameter of the Inhibition Zone

LPEO was tested on five microbial strains, using filter paper diffusion method [46]. Briefly, a suspension of the tested microorganism (10^6^ CFUmL^−1^) was spread on the solid media plates. The paper discs (Whatman No. 1 filter paper, 6 mm diameter) were impregnated with 20 μL LPEO and placed on the inoculated agar. The plates inoculated with bacterial strains were incubated for 24 h at 37 °C. After incubation, diameter of the inhibition zone was measured in millimeters. Each test was performed in triplicates on at least three separate experiments.

#### 3.4.3. Determination of Minimum Inhibitory Concentration (MIC)

MIC values of LPEO against microorganisms were determined by disc-diffusion method [46,47] Sterile filter paper discs were placed on the surface of Petri dishes and impregnated with 20 µL of EO at different concentrations (100.00, 50.00, 25.00, 12.50, 6.25, 3.125, 1.56, 0.78, 0.39, and 0.195 mg/mL) in dimethyl sulfoxide (DMSO). DMSO alone was used as negative control. After staying at 4 °C for 2 h, all Petri dishes were incubated at 37 °C for 24 h. All determinations were performed in triplicates. The minimum inhibitory concentration (MIC) values were determined as the lowest concentration of EOs that inhibited visible growth of the tested microorganism.

### 3.5. Free Radical-Scavenging Capacity

#### 3.5.1. DPPH Radical-Scavenging Assay

The free radical-scavenging activity of LPEO was measured using the stable radical 2,2-diphenyl-1-picrylhydrazyl (DPPH) assay [48]. DPPH was dissolved in ethanol at concentration of 0.1 mmol L^−1^. The absorbance of 2.7 mL DPPH solution and 0.3 mL ethanol was measured as the negative control. A different concentration of the sample solution in ethanol (0.3 mL) was pipetted into a cuvette with 2.7 mL DPPH solution. The resultant solution was incubated for 30 min at room temperature in the dark, and then monitored at 517 nm. The DPPH scavenging activity was expressed according to the following equation:

DPPH scavenging activity (%) = (A_C_ − A_S_)/A_C_ × 100 (1)

where A_C_ is the absorbance of the negative control, and A_S_ is the absorbance containing 0.3 mL sample and 2.7 mL DPPH solution. All samples were analyzed in triplicates, and the results are expressed as the mean ± standard deviation. The scavenging activity was expressed as the 50% inhibitory concentration (*IC*_50_), which was defined as the sample concentration necessary to inhibit DPPH radical activity by 50% after incubation.

#### 3.5.2. ABTS Radical-Scavenging Assay

This method was performed as described by Teles et al. [49], based on the capacity of LPEO to inhibit the 2,2′-azinobis (3-ethylbenzthiazoline-6-sulfonic acid) radical (ABTS). Twenty-five mL of ABTS (7 mM) were added to 440 μL of potassium persulfate (K_2_S_2_O_8_, 140 mM), and the solution was kept in darkness for 12 h at room temperature in order to form the radical. An accurate volume of the solution was diluted in ethanol until an absorbance of 0.70 at 734 nm. Once the radical was formed, 2 mL of ABTS solution were mixed with 100μL of LPEO and the absorbance measured at734 nm. ABTS scavenging effect was calculated using the following equation:

ABTS scavenging activity (%) = (A_C_ − A_S_)/A_C_ × 100 (2)

where A_S_ is the absorbance of the solution when the sample has been added and A_C_ is the absorbance of the ABTS solution as control. The *IC*_50_ was calculated from the graph of scavenging percentage against LPEO concentration. The results are expressed as the mean ± standard deviation.

### 3.6. Cancer Cell Culture

HCT116 colon cancer cells and HepG2 liver cancer cells were purchased from Library of Typical Culture of Chinese Academy of Sciences (Shanghai, China). HCT116 cells were cultured in Dulbecco’s modified Eagle’s medium (DMEM; Hyclone, UT, USA), supplemented with 10% fetal bovine serum (FBS) and 1% penicillin/streptomycin (Hyclone, UT, USA). HepG2 cells were cultured in MEM containing 10% FBS and 1% penicillin/streptomycin (Hyclone, UT, USA). The above-mentioned cells were maintained in 25 cm^2^ cell culture flasks in a humidified atmosphere containing 5% CO_2_ at 37 °C. Cells were fed until 90% confluence and the confluent cells were washed twice with phosphate buffered saline (PBS), treated with 0.25% trypsin (Invitrogen, MA, USA) for about 1 min, and incubated at 37 °C. When the cells were contracted and rounded under the microscope, FBS (Hyclone, UT, USA) containing medium was added, centrifuged at 200× *g* for 3 min, and subcultured at a split ratio of 1:3.

### 3.7. Antiproliferative Activity Test of LPEO

The cell proliferation inhibition rate of LPEO was evaluated by CCK-8 assay [42,43]. LPEO (50 μL) was added to the medium and mixed well. The mixture was diluted in DMSO to prepare solutions at a concentration of 0.5, 0.4, 0.3, 0.2, 0.1, 0.05, and 0.0 μL/mL, respectively. The cells were placed into 96-well plates (3 × 10^3^ cells/well). After 24 h, 100 μL of LPEO at different concentrations was added and continued to incubate for 48 h at 37 °C in a CO_2_ incubator, after which the medium in the 96-well plate was disposed. A 100 μL of CCK-8 test solution (DojinDo, Tokyo, Japan) was added and incubated for 2 h at 37 °C. The optical density (OD) for each well was measured at 450 nm using a microplate reader (BioTek, Winooski, VT, USA). The cell viability rate at different concentrations of LPEO treatment was calculated according to the formula:

Viability rate (%) = (OD_sample_ − OD_blank_)/(OD_control_ − OD_blank_) × 100% (3)

### 3.8. Statistical Analysis

The mean and standard deviation of three experiments were determined. Statistical analyses of the differences between mean values obtained for experimental groups were calculated using IBM SPSS Statistics 23.0. (IBM Corp. Released 2015. IBM SPSS Statistics for Windows, Version 23.0. Armonk, NY, USA). *p* values < 0.05 were regarded as significant, *p* values < 0.01 as very significant and *p* values < 0.001 as highly significant.

## 4. Conclusions

Essential oils are valuable plant extracts used in food, medicine and complementary treatment strategies [41]. The beneficial role of grapefruit EO has been widely reported. However, the bioactivities of grapefruit EO prepared by molecular distillation has not been well studied. Molecular distillation is a very useful technique to separate thermally-sensitive EOs. In our study, molecular distillation was used to remove undesired components from the cold-pressed grapefruit EO to provide light phase EO (LPEO). The chemical composition and antimicrobial activity of LPEO were studied. LPEO showed a wide spectrum of antimicrobial activity against some Gram-positive and Gram-negative microorganisms, with MIC values ranging from 0.78 to 12.50 µL/mL. LPEO might be used as a novel antimicrobial agent in the food industry. The antioxidant activity of LPEO by DPPH and ABTS was obtained with *IC*_50_ values of 22.06 ± 0.92 mg/mL and 15.72 ± 0.32 mg/mL, respectively. An in vitro test showed a dose-dependent antiproliferative activity of LPEO on HepG2 and HCT116 cancer cells. Thus, LPEO may potentially be used as a new complementary anticancer agent. However, this still needs further studies.

## Figures and Tables

**Table 1:** Chemical composition of grapefruit light phase essential oil (LPEO) by GC-MS.

No.	RI^a^	Compounds	Composition (%)
1	938	α-Pinene	0.76
2	956	Camphene	0.01
3	977	Sabinene	0.60
4	985	β-Pinene	0.05
5	992	β-Myrcene	2.16
6	1007	Octanal	0.36
7	1049	Limonene	93.33
8	1053	β-Ocimene	0.02
9	1103	Linalool	0.12
10	1108	Nonanal	0.05
11	1127	*trans*-*p*-Mentha-2,8-dien-1-ol	0.16
12	1137	*cis*-Limonene oxide	0.43
13	1141	*trans*-Limonene oxide	0.33
14	1155	Citronellal	0.04
15	1199	α-Terpineol	0.13
16	1208	Decanal	0.19
17	1251	Carvone	0.41
18	1377	α-Copaene	0.13
19	1388	β-Cubebene	0.14
20	1421	Caryophyllene	0.20
21	1457	Humulene	0.03
22	1482	Germacrene D	0.01
23	1519	δ-cadinene	0.04
24	1566	Caryophyllene oxide	0.04
Total			99.74
Monoterpene hydrocarbons			96.93
Oxygenated monoterpenoids			1.62
Sesquiterpene hydrocarbons			0.55
Oxygenated sesquiterpenes			0.04
others			0.60

RI^a^, retention indices determined on HP-5 column, using the homologous series of n-alkanes (C8–C20).

**Table 2:** The antimicrobial activity of grapefruit light phase essential oil (LPEO).

Bacterial Strain	Diameter of Inhibition Zone (mm)	MIC (µL /mL)
*Bacillus subtilis* (G+)	35.59 ± 1.06 ^a^	0.78
*Staphylococcus aureus* (G+)	24.34 ± 0.52 ^c^	6.25
*Escherichia coli* (G-)	26.86 ± 0.17 ^b^	6.25
*Salmonella typhimurium* (G-)	21.70 ± 0.21 ^d^	12.50
*Pseudomonas aeruginosa* (G-)	8.57 ± 0.13 ^e^	25.00

Disk diameter is 6.0 mm. Zone of growth inhibition values are presented as mean± standard deviation for at least three experiments. Different superscript letters represent the significant differences at *P* < 0.05 according to Tukey’s multiple range test. The scale of zone of inhibition measurement was the following (disk diameter included): ≥20 mm is strongly inhibitory; <20–16 mm is moderately/mildly inhibitory; <15–10 mm is weak inhibitory; <9–7 mm is not inhibitory.
